# Effect of pH and Nanoparticle Capping Agents on Cr (III) Monitoring in Water: A Kinetic Way to Control the Parameters of Ultrasensitive Environmental Detectors

**DOI:** 10.3390/mi11121045

**Published:** 2020-11-27

**Authors:** Chawki Awada, Hassan Traboulsi

**Affiliations:** 1 Department of Physics, College of Science, King Faisal University, P.O. Box: 400, Al-Ahsa 31982, Saudi Arabia; 2 Department of Chemistry, College of Science, King Faisal University, P.O. Box: 400, Al-Ahsa 31982, Saudi Arabia

**Keywords:** trivalent chromium, EDTA, silver nanoparticles, citrate and oxalate capping agents, pH, Raman spectroscopy, SERS, kinetic, Prout–Tompkins model

## Abstract

In this work, we apply surface-enhanced Raman spectroscopy (SERS) to study the kinetics of chromium Cr (III) detection in solution using EDTA and silver nanoparticles (AgNPs). We examine for the first time the effect of pH and nanoparticles’ capping agent on the kinetic mechanism of Cr (III) detection using SERS temporal variations. The full width at half maximum (FWHM) and Raman shift variations show that the mechanism of detection is composed of two steps: a first one consisting of chemical coordination between Cr (III) and AgNPs that leads to exalted chemical and electromagnetic enhancement and the second one is an aggregation process with an important optical enhancement. The obtained results showed that the first step in the detection at lower pH was five times faster than in a basic medium using citrate capped silver nanoparticles (Cit-AgNPs). On the other hand, using a capping agent with dicarboxylate groups such as oxalate (Oxa-AgNPs) led to an important enhancement in SERS detection signal (more than 30 times) compared with Cit-AgNPs, although the detection kinetic’s mechanism was slower.

## 1. Introduction

Surface-enhanced Raman spectroscopy (SERS) is an ultrasensitive technique that can be used for trace quantities detection of various analytes by using plasmonic metallic nanostructures such as silver and gold nanoparticles [1,2,3,4,5]. A successful SERS signal requires good molecular interactions with the nanoparticles so that the proximity time of the analytes from the surface is long enough to allow an enhanced and stable detection process [6]. Molecules exhibiting good affinity to the surfaces will possess longer residence times at the interface. Thus, the kinetics of molecules’ detection is closely dependent on mass transport to the metal surface and intermolecular interaction energetics [7]. Colloidal nanostructures such as silver nanoparticles (AgNPs) represent an important way to decrease the fluctuations of SERS intensity and overcome the lack of reproducibility, mainly comparing to the rough surface [8]. However, the contact between target analytes and the surface of metal nanoparticles required to reach the highest SERS enhancement is restricted to molecules with a good chemical affinity toward the surface of nanoparticles. Thus, strategies to control the surface chemistry to enhance the interactions between analyte and metal nanoparticles have been investigated by modifying the charge on the surface, changing the metal capping or using specific guest–host interaction to enhance SERS signal [9,10]. Functionalizing metal nanoparticles with an active agent, which is monitored by applying external factors such as pH, heat or even ionic strength, is known as an approach to confine molecules closer to the surface of nanoparticles and thus increases the SERS signal [11]. Álvarez-Puebla et al. reported gold nanoparticles coated by poly-(N-isopropylacrylamide), which is a thermally responsive polymer. In the lattice study, the authors showed that when the temperature was greater than 60 °C, the coating network collapsed, making the analyte closer to the surface of nanoparticles, causing an important enhancement of the Raman signal [12]. In another work, Furini et al. have synthesized AgNPs activated with poly(allylamine) hydrochloride (PAH) shell that helped to enhance the SERS signal due to analyte trapping by swelling and deswelling according to the value of pH in the medium [13]. SERS signal depends on surface plasmon resonance (SPR) and undergoes an important enhancement when the label analytes are present at the junction of aggregates with multiple nanoparticles [14]. This is induced by the plasmon coupling between the nanoparticles when they are very close from each other, leading to a great electromagnetic (EM) enhancement at aggregation sites as SERS “hot spots [15].” According to reported calculations, closely spaced nanoparticles could generate an important enhancement at the junctions of two or more nanoparticles, and the greatest enhancement of SERS signal is likely obtained at the fractal space in aggregated nanoparticles [16,17]. Therefore, the aggregation behavior of AgNPs plays an important factor in SERS technique. It is time-dependent and very sensitive towards many factors, including solution chemistry (e.g., ionic strength, pH, electrolyte composition) and nanoparticle coating layer [18]. The local electrical field enhancement distribution study was reported on a nanostructured gold film by using functionalized tip-enhanced Raman scattering, the distance between the hotspot and the probing molecule affected strongly the enhancement of Raman intensity and shift variation. In the latter, experimental and theoretical study confirmed the presence of two attenuation regions, one is short (when the molecules is placed near the nanoparticles within 0–4 nm ranges and the second one is slower, it is between 4 nm and 150 nm [19]. The size of the nanoparticles could also affect the SERS signal. Indeed, it has been shown that an increase in size of the metal nanoparticles induces a greater enhancement detection for each analyte used [20]. Recently, we have reported the first kinetic studies on chromium (III) detection in aqueous solution using citrate capped silver nanoparticles (Cit-AgNPs), ethylenediaminetetraacetic acid (EDTA) and surface enhanced Raman spectroscopy (SERS) technique) [21]. This study was conducted at pH 8.1, and the EDTA chelator appeared to have an important effect on the mechanism and kinetics of Cr (III) detection as well as on the stability and the enhancement of the SERS signal. We have suggested in the latter work that the chemical coordination, which occurs on the surface of AgNPs with citrate or EDTA molecules, could be considered as an autocatalytic reaction for particle aggregations and hence the formation of hot spots for SERS detection. An important difference has been observed in the kinetics and mechanisms of chromium (III) detection depending on the form of EDTA that is present in the medium. As a free chelator that will coordinate with Cr (III) in situ or as a formed complex [Cr(EDTA)]-, the kinetic that raised from the temporal SERS signal was different. When added as a free ligand to the medium in the presence of chromium (III) and citrate capped AgNPs, the monitoring of Cr (III) has led to an important enhanced and stable SERS signal.

Continuing our investigation of the developed process, we report here a complementary study, where we examine the effect of pH and the capping agent on the enhancement and kinetics of Cr (III) detection. By using the SERS technique, we extracted from the Raman spectra the time-dependent intensity, Full width at half maximum (FWHM) and Raman shift. Time-dependent intensities are well-matched with a Prout–Tompkins equation in order to extract the limited rate constant of the kinetic mechanism. Acidic pH shows faster kinetic detection than the basic one. FWHM and Raman shift variation confirm the presence of two steps: chemical coordination dominated by a chemical enhancement and the second one an aggregation with a pure optical enhancement. On the other hand, the capping effect was studied by using oxalate as AgNPs capping molecules. From SERS intensity, consequently, we observed stronger enhancement than in the case of the citrate capping. In parallel, scanning electronic microscopy (SEM) measurements and UV-visible absorption were performed to observe the aggregation features after changing the pH of the medium and the capping of silver nanoparticles.

## 2. Experimental

### 2.1. Reagents and Solutions

Sodium borohydride (NaBH_4_), disodium oxalate (Na_2_C_2_O_4_), trisodium citrate (Na_3_C_6_H_5_O_7_), citric acid (C₆H₈O₇), sodium carbonate (Na_2_CO_3_), chromium (III) nitrate nonahydrate (Cr (NO_3_)_3_∙ 9H_2_O and silver nitrate (AgNO_3_) were purchased from Sigma Aldrich. Fresh water for solution preparations was obtained by distillation and purification using a Milli-Q system (Millipore, Milford, MA, USA).

### 2.2. Preparation of Citrate or Oxalate Capped Silver Nanoparticles (AgNPs)

Before synthesis, all the glassware was washed thoroughly with water and dried with air. The citrate capped silver nanoparticles (Cit-AgNPs) were prepared according to reported work with slight modifications [21]. Briefly, 1 mL of 44 mM trisodium citrate or disodium oxalate is added to ~50 mL of 1.1 mM boiling solution of silver nitrate under stirring on a hotplate. This step was followed by the dropwise injection of ice-cold freshly prepared aqueous of NaBH_4_ solution (1 mL, 0.03 M) using a syringe where a pale-yellow color has marked the formation of Cit-AgNPs. The pH was measured and found equal to 8.1, then adjusted to 3.6 using a 1 M solution of citric acid or to 9.6 using a 1 M solution of sodium carbonate. The same method was used for the synthesis of oxalate capped silver nanoparticles, where disodium oxalate was used instead of trisodium citrate. However, due to the instability of the prepared nanoparticles in these conditions, the pH was adjusted directly to 9.6 using the 1 M solution of sodium carbonate. In all the synthesis processes, the dispersions were kept under stirring for 30 min at room temperature to assure completion of the reactions.

### 2.3. Scanning Electron Microscopy (SEM)

For sample preparation, few drops of the different nanoparticles in mixture with Cr (III) + EDTA were deposited on a clean copper metal substrate disc and kept for water evaporation before conducting the SEM measurements. A Jeol, JSM 7000 series scanning electron microscope was used to perform the morphological micrographs of AgNPs at 15.0 kV scan voltage.

### 2.4. UV-Visible Spectroscopy

The different samples of citrate or oxalate capped silver nanoparticles (as-synthesized AgNPs, and the mixtures with Cr (III) + EDTA) were characterized by UV-visible spectroscopy. The different mixtures were prepared 2 h before the UV-visible absorption measurements. The UV-2600 spectrophotometer (Shimadzu, Tokyo, Japan) was used to perform the spectra. Water was used as a blank to record the spectra, and the obtained data were plotted using Origin software.

### 2.5. SERS Measurements

Fresh solutions of the mixture EDTA + Cr (III) (prepared in equal ligand to metal mole ratio in water), was mixed with silver nanoparticles (in the ratio 1:4; v:v; mixture: AgNPs) just before SERS measurements. The concentration of EDTA or Cr (III) after mixing with AgNPs was 5 × 10^−4^ M. The mixing time with AgNPs was used as the starting point for our kinetic studies. A clean quartz cell was completely filled with the mixture, sealed and put under Raman laser to start the SERS measurements (Supplementary Materials Figure S1). Raman spectra were collected using LabRam HR evolution spectrometer (Horiba Scientific, Kyoto, Japan) in a backscattering geometry with a spectral resolution of 1.2 cm^−1^ at ambient temperature. A He–Ne laser of λ = 632.8 nm and a power level of 2 mW were employed. The lower power density was chosen in order to prevent any thermal effect. An objective of 10× with a numerical aperture of N.A. 0.25 was used. The latter offers the capability of a large excitation of a high number of molecules in solution. The Raman spectra were collected from sample solutions placed in the quartz cuvette that can let the light being transmitted into the solution without any considerable loss of intensity. In this setup, the laser focus was fixed in order to avoid variations in the excitation volume, which could generate fluctuations in Raman signal intensity.

## 3. Results and Discussion

### 3.1. Effect of pH

**Figure 1:**
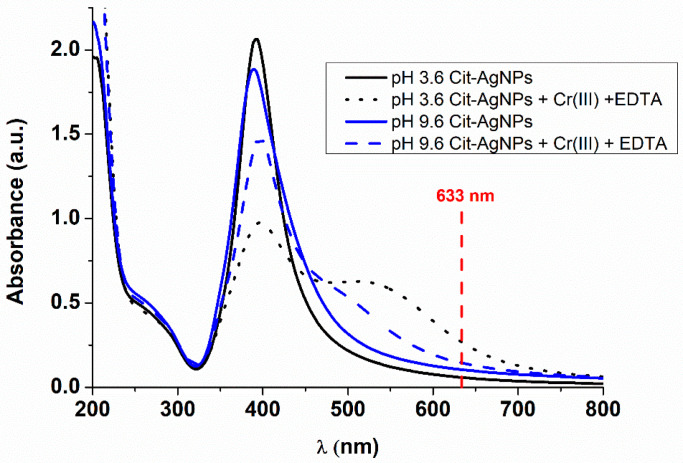
UV-visible spectra of citrate capped silver nanoparticles. Black solid line: Cit-AgNPs at pH = 3.6; black dot line: Cit-AgNPs in presence of the mixture Cr (III) + EDTA at pH = 3.6; blue solid line: Cit-AgNPs at pH = 9.6; blue dashed line: Cit-AgNPs in presence of the mixture Cr (III) + EDTA at pH = 9.6.

The dependence of AgNPs surface plasmon resonance (SPR) on different parameters such as size, shape and chemical environment allows the study of the physical and chemical modifications, aggregation and kinetic parameters that are associated with the colloidal system [22]. The idea from the UV-visible study is to show the effects of the different analytes on the aggregation of AgNP_S_ and thus on their plasmon. The colloidal dispersions were diluted five times as the silver nanoparticles exhibited high absorbance in UV. The UV-visible absorption spectra of the citrate-coated silver nanoparticles Cit-AgNPs at pH = 3.6 and pH = 9.6 are presented in Figure 1. Before adding the mixture Cr (III) + EDTA, the absorbance peaks of citrate capped AgNPs were centered at around 393 and 389 nm for acidic and basic pH, respectively. This result indicates a smaller average of nanoparticle sizes at higher pH, which is in agreement with the literature [23]. On the other hand, it is known that silver nanoparticles, in the absence of analytes, will be existing in the colloidal system as single particles and dimer aggregate, explaining the position of the maximum absorption at around 390 nm (Figure 1, solid spectra).

**Figure 2:**
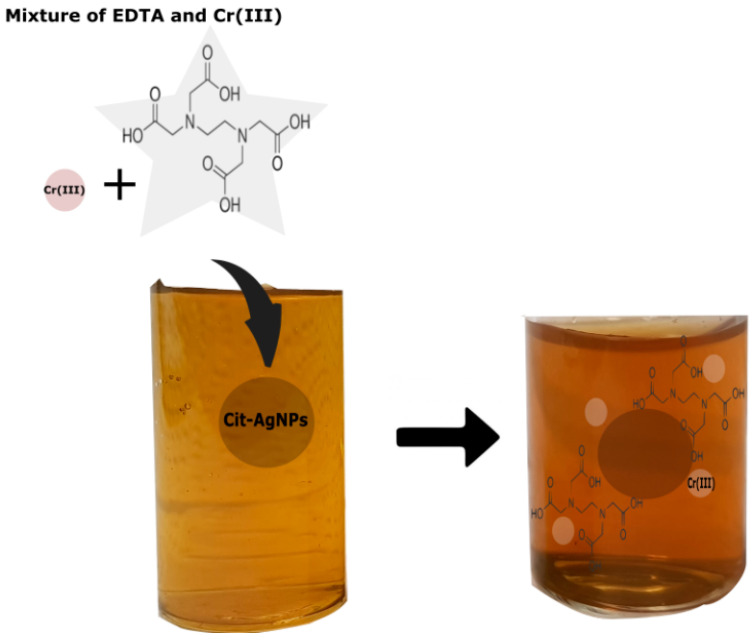
Speculation of ethylenediaminetetraacetic acid (EDTA) + Cr (III) adsorption on Cit-AgNPs. The color of the solution changes to red, indicating a shift of AgNPs plasmon.

When the mixture of Cr (III) + EDTA is added to the silver nanoparticles, the coordination between Cr (III), EDTA and Cit-AgNPs will occur in situ on the surface as speculated in Figure 2. The color of the dispersion changed from yellow to red, indicating a shift in the plasmon (Figure S2). The aggregation process is known to be fast, and the kinetic cannot be monitored by UV-visible spectroscopy. For example, Xu et al. have shown that the plasmon shift due to aggregation of tartrate capped silver nanoparticles is fast and dependent on the concentration of Cr (III) [24].

After 2 h of mixing the analytes with Cit-AgNPs, the UV-visible absorption spectra were recorded, and a plasmon shift to a higher wavelength was observed (Figure 1, dotted and dashed lines). Indeed, the addition of Cr (III) + EDTA should lead to more aggregations of AgNPs, and consequently, the particles become closer to each other, which explains the presence of two resonances at around 380 nm and 520 nm, attributed to the dipolar and quadripolar mode, respectively. The surface plasmon absorption of silver nanoparticles is very sensitive to the variations in the surroundings and is strongly influenced by the physical and chemical modifications on the particle surfaces. Liu et al. have studied the adsorption of different cations on the surface of noncoated AgNPs [25]. The authors showed that in the presence of certain amounts of transition metal cations, the aggregation of silver nanoparticles occurred. In addition, a new band centered near 510 nm in the absorption spectra of silver nanoparticles appeared. The peak position of the new band depended on nature as well as the concentration of the used metal cations. The authors have concluded that the band near 510 nm is attributed to the double effects of the adsorption of metal cations onto the surfaces of silver nanoparticles and the aggregation of silver nanoparticles. Our UV-visible data show that the plasmon shift is more observed in the case of the acidic medium, indicating that the adsorption and the aggregations occur more on the surface of the silver nanoparticles at pH 3.6 compared with the basic medium. This observation could be explained by the hydrolysis of Cr (III) at higher pH, leading to the formation mainly of Cr(OH)_3_. However, at pH 3.6, the most abundant species of Cr (III) will be Cr^3+^, which could be easily adsorbed on the negatively charged Cit-AgNPs [26].

**Figure 3:**
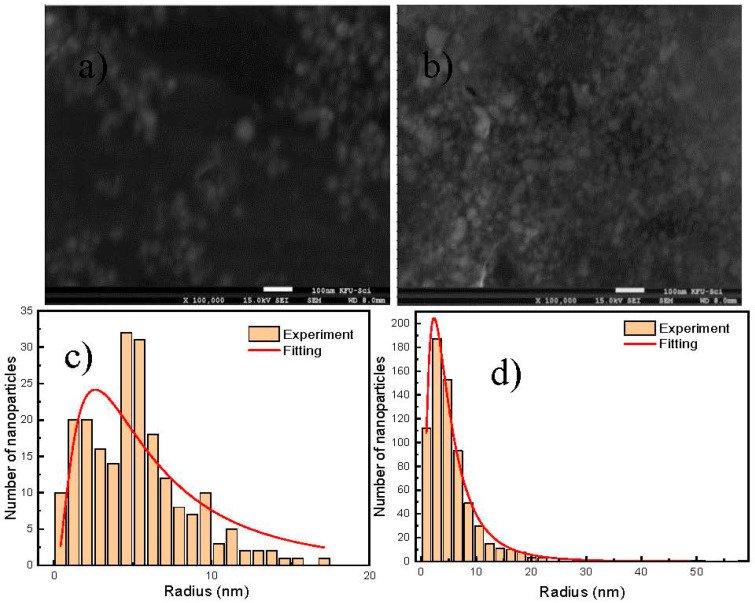
SEM images of Cit-AgNPs before mixing with EDTA + Cr (III) at different pH and size distribution of AgNPs taken from SEM image, the size distribution was fitted by a lognormal function $y=y_{0}+\frac{A1}{\sqrt{2\pi \sigma x}}e^{\frac{-{\left[lnx/r\right]}^{2}}{2\sigma 1^{2}}}$ (**a**) SEM image for pH = 3.6. (**b**) SEM image for pH = 9.6. (**c**) The average of size extracted for pH = 3.6 with its deviation is 12 nm (0.9 nm), adj. R-squared 0.852. (**d**) The average of size extracted for pH = 9.6 with its deviation is 8 nm (0.7 nm), adj. R-squared 0.998.

Figure 3a–d shows the SEM images of Cit-AgNPs before adding the mixture Cr (III) + EDTA for the two different values of pH 3.5 and 9.6 with their distribution of size. We can observe a decrease in the average size from 12 nm ± 0.9 nm to 8 nm ± 0.7 nm when the pH increases from 3.6 to 9.6. This can explain the redshift of the plasmon peak located around 390 nm of the acidic medium relative to the basic one (see Figure 1).

**Figure 4:**
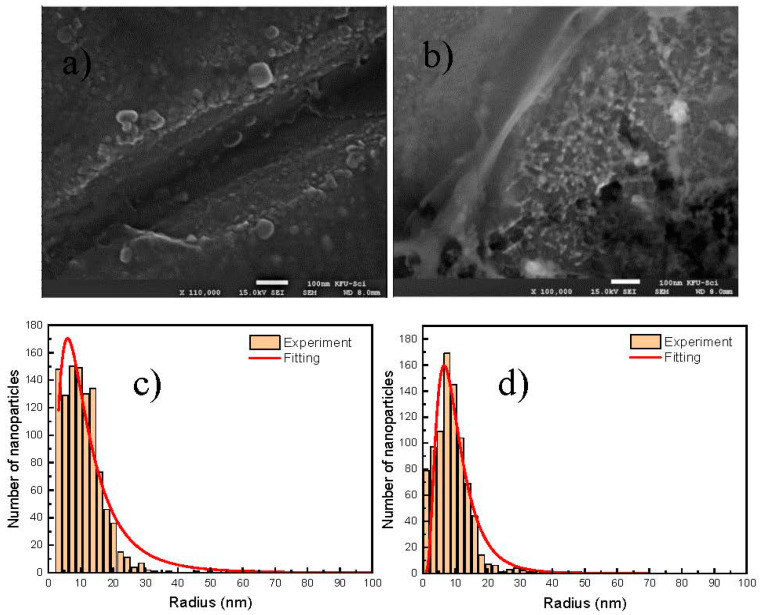
SEM images of Cit-AgNPs mixed with EDTA + Cr (III) at different pH and size distribution of AgNPs taken from SEM image, the size distribution was fitted by a lognormal function $y=y_{0}+\frac{A1}{\sqrt{2\pi \sigma x}}e^{\frac{-{\left[lnx/r\right]}^{2}}{2\sigma 1^{2}}}$. (**a**) SEM image for pH = 3.6. (**b**) SEM image for pH = 9.6. (**c**) The average of size extracted for pH = 3.6 with its deviation is 21 nm (0.7 nm), adj. R-squared 0.931. (**d**) The average of size extracted for pH = 9.6 with its deviation is 18 nm (0.5 nm), adj. R-squared 0.912.

**Figure 5:**
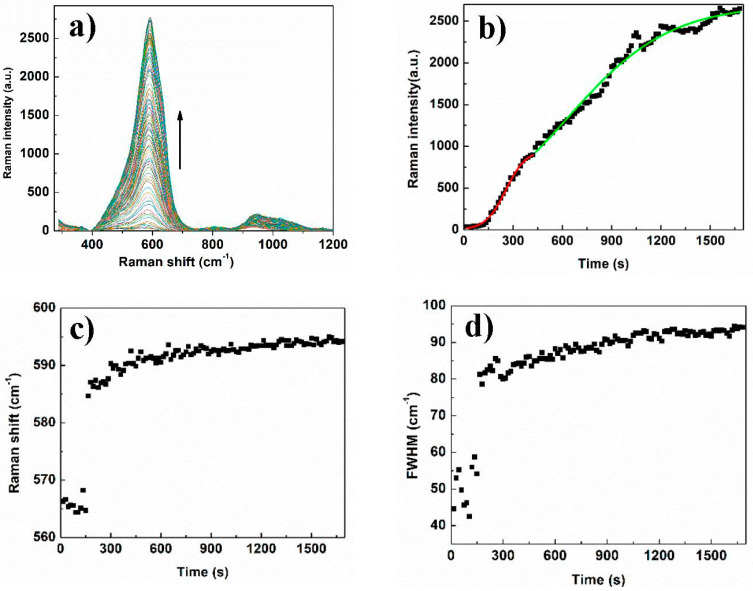
(**a**) Time-dependent Raman spectrum of Cr (III) + EDTA in presence of Cit-AgNPs at pH = 3.6. (**b**) plotting and fitting the maximum of Raman intensity versus time by using Prout–Tompkin equation. Between 0 and 400 s, *k* = 0.98 min^−1^, adj. R-squared 0.99 and between 400 s and 1800 s, *k* = 0.19 min^−1^, adj. R-squared 0.98. (**c**) Raman shift versus time of Cr (III) + EDTA in presence of Cit-AgNPs at pH = 3.6 (**d**) FWHM variation of the Raman peak versus time of Cr (III) + EDTA in presence of Cit-AgNPs at pH = 3.6.

**Figure 6:**
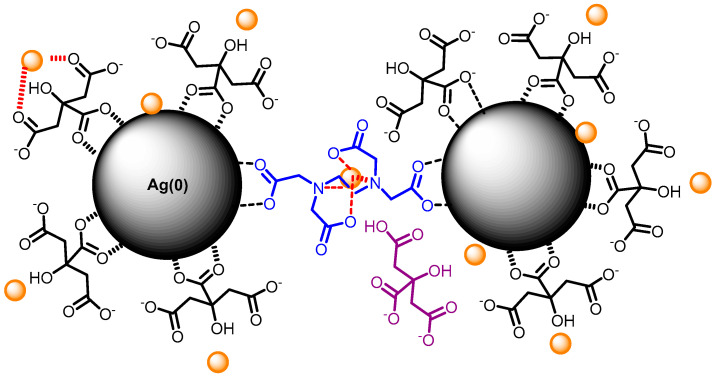
A speculated scheme of Cr (III) coordination with citrate and EDTA on AgNPs surface. Small orange spheres represent Cr (III), molecules in blue represent the EDTA chelator in complex with Cr (III), molecules in black color represent the citrate capping the AgNPs, molecule in purple color represents citrate after being released from AgNPs surface.

Figure 4a–d shows the SEM images of Cit-AgNPs after adding the mixture Cr (III) + EDTA for the two different values of pH 3.5 and 9.6 with their distribution of size. We can see that the aggregate and the agglomeration features of silver nanoparticles change from the lowest to the highest pH. In an acidic medium, we can observe large and small particles, which can explain the presence of quadripolar and dipolar modes of plasmons in the UV-visible absorption spectra that are located, respectively, at 380 and 520 nm. However, by increasing the pH to 9.6, we can see less agglomeration of particles. This can explain the dominance of the quadripolar mode located at 390 nm compared to the dipolar mode (Figure 1). The SERS experiments were done in solution in order to examine the different interactions that can occur between the different components of the studied system. Initially, the time-dependent SERS measurements were performed to study the effect of pH on the kinetics of Cr (III) detection and the SERS enhancement using EDTA and citrate-capped silver nanoparticles. The SERS signals at pH 3.6 indicate an increase in the Raman intensity of the vibrational mode, where the maximum intensity varies from 565 to 595 cm^−1^ (Figure 5). The maximum intensity of each peak (Figure 5b) was extracted by fitting all the spectra shown in Figure 5a. We have attributed this signal to the Raman chromium–oxygen and chromium–nitrogen stretching bands with citrate and EDTA molecules) [21]. Additionally, a large band centered around 1000 cm^−1^ could be attributed to the C-H in-plane bending of citrate and EDTA [27]. The chelator EDTA can also contribute to the formation and stabilization of further aggregates to generate additional hot spots for SERS signal enhancement. It is worth noting that at pH around 3.6, only one carboxylic group of the citric acid is significantly deprotonated as the dissociation constants of citric acid are pk_1_ = 3.13, pk_2_ = 4.74, and pk_3_ = 6.40 [28]. On the other hand, EDTA is an hexaprotic weak acid that can form complexes 1:1 with metal cations and will have four deprotonated carboxylic acids at pH 3.6. This flexible molecule could coordinate with the surfaces of Cit-AgNPs and progressively with Cr (III) (as speculated in Figure 6), considering that the formation of the complex between EDTA and Cr (III) is slow [28].

This construction in solution would allow the proximity between the silver nanoparticles, which is confirmed by the SERS enhancement in the second step of the detection mechanism.

We have carefully examined the Raman peak shifts and the FWHM variation that occurs during the SERS measurements. A Raman shift (from 565 to 595 cm^−1^) and an increase of FWHM are obtained between 0 and 400 s and stabilize after this (Figure 5c,d). This observation indicates that the coordination with Cr (III) is mainly occurring within the first 400 s leading to the formation of more aggregated hot spots during the rest of the measurement, which is associated with an electromagnetic enhancement. It is worth noting that electromagnetic enhancement is known to be more displayed than chemical enhancement, which is in agreement with our observation [29]. Indeed, chemical enhancement is a weak effect and likely originated from a combination of resonances between the metal–molecule complex, electron charge transfer between molecules and the plasmonic surface [30,31]. Based on the proposed nucleation site buildup kinetic model in our reported work, we can also suggest here that the coordination between Cit-AgNPs with the mixture Cr (III) and EDTA can lead to the formation of aggregates that are considered as hotspots for SERS signal enhancement [21]. Thus, fitting the temporal response of the SERS spectral features taken from the maximum of intensities was done on the fast step (0 to 400 s) and the slow step (400 to 1800 s) using the Prout–Tompkins equation [32].

The rate equation of a first-order autocatalyzed reaction [33] is given by Equation (1):

(1) $$\frac{d\theta }{dt}=k_{b}\left(1-\theta \right)$$

where *θ* is the conversion fraction, *t* is the time of reaction, and $k_{b}$ is the conversion-fraction-dependent rate constant. In autocatalyzed reactions, $k_{b}$ is assumed to be a linear function of θ; therefore, $k_{b}=k\theta$, Where *k* is the conversion-fraction-independent rate constant.

The rate of Equation (1) can be rewritten as in Equation (2):

(2) $$\frac{d\theta }{dt}=k\theta \left(1-\theta \right)$$

**Figure 7:**
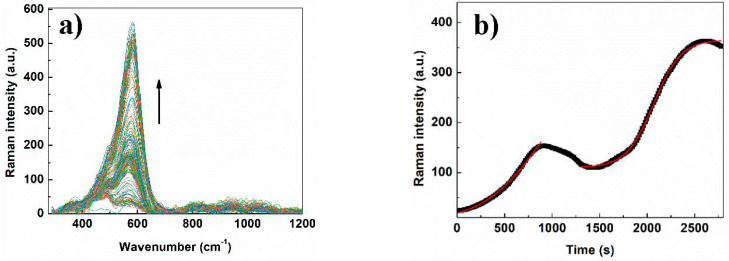
(**a**) Time-dependent Raman spectrum of Cr (III)+EDTA in the presence of Cit-AgNPs at pH = 9.6. (**b**) plotting and fitting the maximum of Raman intensity versus time by using Prout–Tompkins equation. First adjustment between 0 and 1000 s, *k* = 0.18 min^−1^, Adj. R-squared 0.995. Second one between 1300 s and 2750 s, *k* = 0.42 min^−1^, Adj. R-squared 0.999.

Integrating and rearranging Equation (2) will lead to Equation (3) as follows:

(3) $$\theta ={\left(1+e^{-k\left(t-t_{0}\right)}\right)}^{-1}$$

where *t*_0_ is an integration constant. By adjusting the temporal curve of the Raman feature at the maximum intensities as per Equation (3), the rate constant of the slowest step (400 to 1800 s) was calculated and was equal to 0.19 min^−1^. As the slowest step is the rate-determining step of the process, we can consider that the rate constant of this SERS kinetic study is equal to 0.19 min^−1^ at pH = 3.6. Interestingly, the obtained rate constant of the first step (0 to 400 s) of the detection process in these conditions is equal to 0.99 min^−1^ indicating a fast step of Cr (III) coordination on Cit-AgNPs. In order to investigate further the effect of pH, the SERS study was also performed at pH 9.6, where the time-dependent Raman spectra are presented in Figure 7a.

The temporal response taken from the maximum of intensities of the SERS spectra is presented in Figure 7b, where the treatment of the data could be divided into two zones. The first one is located between 0 and 1000 s, where one can see an increase of Raman intensity followed by a drop in the signal. Fitting of this part using the Prout–Tompkins equation leads to the calculation of a first-rate constant equal to 0.18 min^−1^. Surprisingly, this constant is equal to the value obtained in our reported work about the detection of Cr (III) by Cit-AgNPs at pH 8.1 in the absence of EDTA) [15]. This result could allow us to suggest that at this stage, the EDTA is not significantly involved in the coordination of Cr (III) and thus in SERS signal enhancement. The second zone, which is located after 1500 s, comprises the typical increase of the SERS signal that was observed in our reported work after adding EDTA to the Cr (III)-Cit-AgNPs. Fitting the second part of the data using the Prout–Tompkins equation will lead to the obtention of a detection rate constant equal to 0.42 min^−1^ indicating a faster increase in the SERS signal when EDTA is involved in the mechanism. This value was close to the one obtained after adding EDTA to the Cr (III)-Cit-AgNPs at pH = 8.1 (*K* = 0.49 min^−1^), which indicates that EDTA is mainly involved in the second step of the detection mechanism [21]. On the other hand, comparing the rate constant values of the first step at acidic and basic pH reveals that the process is almost 5 times faster at pH 3.6. This observation could be explained by the effect of pH on the hydrolysis of chromium (III). Indeed, at pH 3.6, chromium will be present as Cr^3+^, which can be adsorbed faster on the surface of Cit-AgNPs, leading to a faster process of coordination. However, the hydrolysis of Cr (III) will lead to slower adsorption and thus slower nucleation step for nanoparticle aggregations. Additionally, the examination of the Raman shift and the FWHM (Figure S3a,b) indicates a fluctuation with time mainly before 1500 s, showing the instability of Cr (III) coordination at this pH value before the involvement of EDTA in the process. However, after the involvement of EDTA, the colloidal system at pH 9.6 showed the highest stability among the examined pH where no precipitation was observed after 24 h (Figure S4). It is worth noting that once the nanoparticles are formed, the pH value does not affect their sizes. However, the pH may alter the double layer properties and thus the zeta potential making the nanoparticles more stable at a specific pH [34]. Thus, at basic pH, the complexes around the nanoparticles are negatively charged, which contributes to the stabilization of the colloidal system and avoids the precipitation of AgNPs.

### 3.2. Effect of Capping

**Figure 8:**
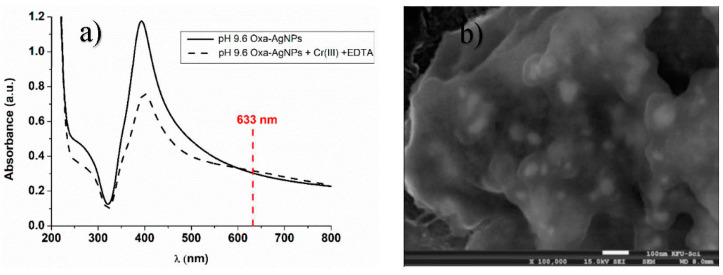
(**a**) UV-visible spectra of oxalate capped silver nanoparticles. Black solid line: silver nanoparticles at pH = 9.60 (Oxa-AgNPs); black dashed line: Oxa-AgNPs in the presence of the mixture Cr (III)-EDTA. (**b**) SEM images of Oxa-AgNPs mixed with EDTA + Cr (III) at different pH = 9.6.

After examination of the pH effect on the SERS detection of Cr (III), we have considered the study of the silver nanoparticles’ capping agent. Thus, oxalate capped silver nanoparticles Oxa-AgNPs were prepared at pH 9.6 as a basic pH was required for nanoparticle stability purposes with this capping agent. The colloidal dispersion of Oxa-AgNPs appears to have a cloudy color, which indicates the formation of aggregations after the preparation of the nanoparticles (Figure S5). It is worth noting that the dicarboxylic oxalate will afford fewer charges around the particles compared with the citrate molecules, which explains the formation of bigger aggregations. The UV-visible spectra of oxalate capped silver nanoparticles are presented in Figure 8a, where the baseline is high due to the light scattering indicating bigger aggregations of nanoparticles. After adding the mixture Cr (III) + EDTA, a flat plasmon resonance between 550 and 700 nm was observed. This flatness in the spectrum could be attributed to Mie scattering originated from the formation of larger size particles or agglomeration, as also confirmed by SEM (Figure 8b) [35].

Oxalate has two carboxylate groups that will be coordinated with AgNPs. The SERS measurements in the presence of the mixture Cr (III) + EDTA were performed as described previously, and the time-dependent Raman spectra are presented in Figure 5a.

**Figure 9:**
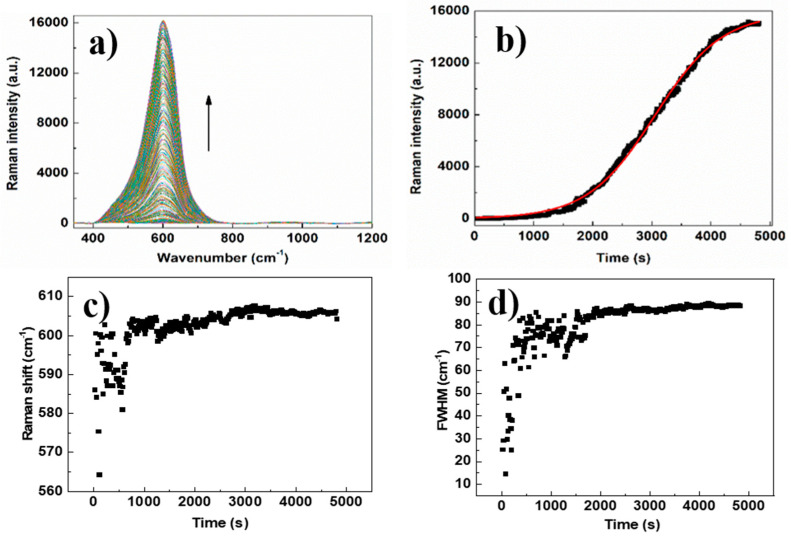
(**a**) Time-dependent Raman spectrum of Cr (III) + EDTA in the presence of Oxa-AgNPs at pH = 9.6. (**b**) Plotting and fitting the maximum of Raman intensity versus time by using Prout–Tompkins equation. *k* = 0.1 min^−1^, Adj. R-squared 0.998. (**c**) Raman shift versus time of Cr (III)+EDTA in the presence of Oxa-AgNPs at pH 9.6. (**d**) FWHM variation of the Raman peak versus time of Cr (III) + EDTA in the presence of Oxa-AgNPs at pH 9.6.

The temporal response taken from the maximum of intensities of the SERS spectra is presented in Figure 9b. Interestingly, an important enhancement of the SERS signal was observed in this case compared with the citrate capped silver nanoparticles. Additionally, we can observe a gradual increase in the SERS signal that follows the Prout–Tompkins model. A greater Raman shift (from 560 to 610 cm^−1^) and an increase of FWHM are obtained between 0 and 1000 s and appear to be stabilized after this (Figure 9b,d). Based on this observation, we can propose that the coordination with Cr (III) is occurring mainly with EDTA and oxalate on AgNPs in the first 1000 s leading to the formation of more aggregated hot spots during the rest of the measurement which is associated with an electromagnetic enhancement. The electromagnetic enhancement is much more displayed (compared with Cit-AgNPs), and it is several orders of magnitude stronger than the chemical enhancement. Thus, the fitting using Prout–Tompkins model was perfectly done on the combination of the two modes of enhancement with an adj. R-squared of 0.998. The comparison of the SERS results obtained with Oxa-AgNPs and Cit-AgNPs shows clearly the effect of the capping agent on Cr (III) monitoring in solution using our technique. The rate constant we obtained in this process *K* = 0.1 min^−1^ indicates a slower kinetic compared with Cit-AgNPs, which could be due to the bigger size of nanoparticles capped with oxalate. Additionally, the lower affinity of Cr (III) toward the dicarboxylic oxalate compared with the tricarboxylic oxalate could explain the lower adsorption of Cr (III) on AgNPS and thus the slower kinetic of SERS signaling.

**Figure 10:**
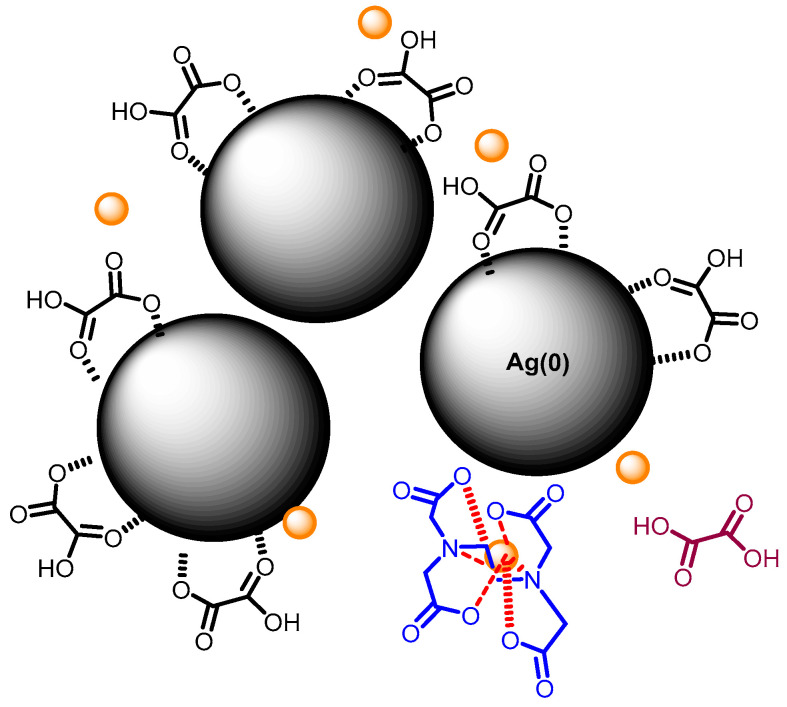
A speculated scheme of Cr (III) coordination with oxalate and EDTA on AgNPs surface. Small orange spheres represent Cr (III); molecules in blue represent the EDTA chelator in complex with Cr (III); molecules in black color represent the oxalate capping the AgNPs; molecule in purple color represents oxalate after being released from AgNPs surface.

The capping agents with dicarboxylate groups such as oxalate agents would be coordinated with the surface of AgNPs and less available for Cr (III) complexation. Thus, more EDTA molecules will be involved in the coordination of Cr (III) on AgNPs, leading to a better signal enhancement (as speculated in Figure 10). The mixed vibration mode chromium–oxygen and chromium–nitrogen is more displayed in this case due to a greater number of EDTA molecules that are in coordination with Cr (III). This could explain the larger FWHM and Raman shift variations compared with the value observed with Cit-AgNPs at the same pH (Figure 9c,d and Figure S3).

## 4. Conclusions

In the present work, we have explored the effect of pH and the nanoparticle capping agent on the detection of Cr (III) in water using the EDTA and SERS techniques. We have observed that when using citrate capped silver nanoparticles, the first step of the detection process was faster at acidic pH compared to basic pH. This result indicates the effect of cation hydrolysis on the adsorption and the coordination with citrate and EDTA. Additionally, we have shown that using an oxalate capping agent with silver nanoparticles has an important effect on the enhancement of the SERS signal of Cr (III) detection. Indeed, oxalate has two carboxylic groups attached to the surface of AgNPs, and in this way, does not contribute significantly to Cr (III) coordination. In parallel, EDTA could coordinate to Cr (III) on the surface, leading to a greater SERS enhancement. This work would pave the road toward the study of different host–guest interactions with SERS technique at different pH and using different nanoparticles capping agent.

## Supplementary Materials

The following are available online at https://www.mdpi.com/2072-666X/11/12/1045/s1, Figure S1: Kinetic experiment on Cr (III) monitoring in solution using EDTA, AgNPs and SERS technique. Figure S2: Photograph of citrate capped silver nanoparticles at pH 3.6 in the absence and in the presence of Cr (III) + EDTA just before SERS measurements. Figure S3: Raman shift and FWHM variation versus time of Cr (III) + EDTA in the presence of Cit-AgNPs at pH 9.6. Figure S4: Photograph of citrate capped silver nanoparticles at different pH. Figure S5: Photograph of Oxalate capped silver nanoparticles at pH 9.6. S1: Estimation of the silver nanoparticles concentration.
