# Supplementary material for: Effect of pH and Nanoparticle Capping Agents on Cr (III) Monitoring in Water: A Kinetic Way to Control the Parameters of Ultrasensitive Environmental Detectors
Source: Micromachines (Basel). 2020 Nov 27;11(12):1045. doi: 10.3390/mi11121045 (PMC7760521; doi:10.3390/mi11121045)
Supplement: Supplementary file 1 [file micromachines-11-01045-s001.pdf]

micromachines

# Supplementary Materials: Effect of pH and Nanoparticle Capping Agents on Cr (III) Monitoring in Water: A Kinetic Way to Control the Parameters of Ultrasensitive Environmental Detectors

Figure S1 describes the conduction of SERS kinetic studies in a quartz cell. A clean quartz cell was completely filled with the mixture, sealed and put under Raman laser to start the SERS measurements.

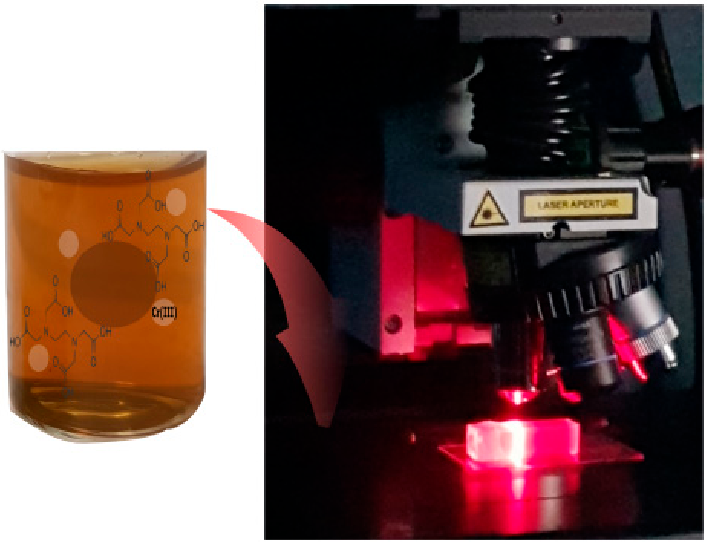

**Figure S1.** Kinetic experiment on Cr (III) monitoring in solution using EDTA, AgNPs and SERS technique.

Figure S2 shows the color change immediately of Cit-AgNPs upon adding the mixture of Cr (III) + EDTA to the silver nanoparticles indicating a plasmon shift.

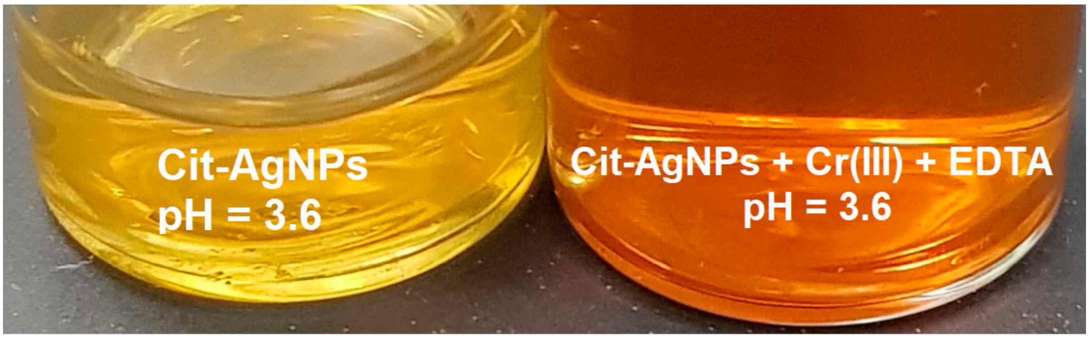


**Figure S2.** Photograph of citrate capped silver nanoparticles at pH 3.6 in absence and in presence of Cr(III) + EDTA just before SERS measurements.

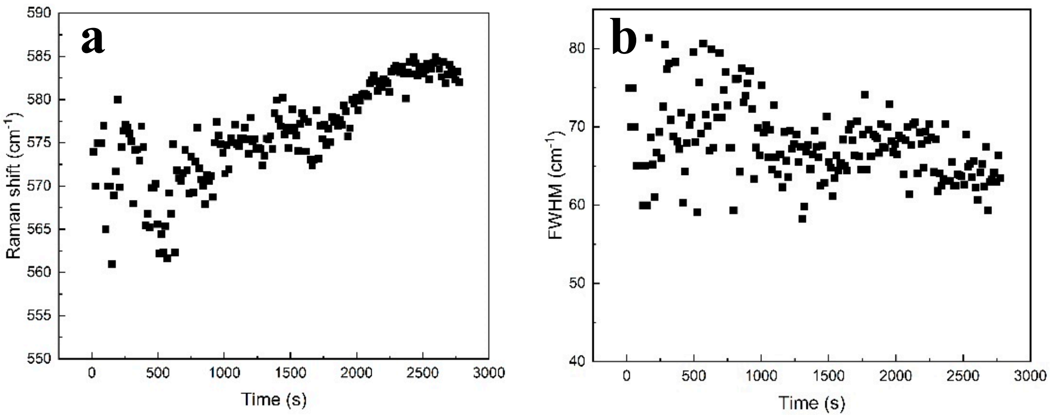


**Figure S3.** (a) Raman Shift versus time of Cr(III)+EDTA in presence of Cit-AgNPs at pH 9.6 (b) FWHM variation of the Raman peak versus time of Cr(III)+EDTA in presence of Cit-AgNPs at pH 9.6.

At basic pH, the fluctuation in Raman shift and FWHM are clearly observed (Figure S2) mainly in the first step, where EDTA was not yet involved in the mechanism of coordination with Cr(III). This fluctuation could be explained by the desorption of Cr(III) from the citrate coordination cavity. After 1500 seconds, less fluctuations are observed which due to the stable coordination with Cr(III) on the surface of Cit-AgNPs.

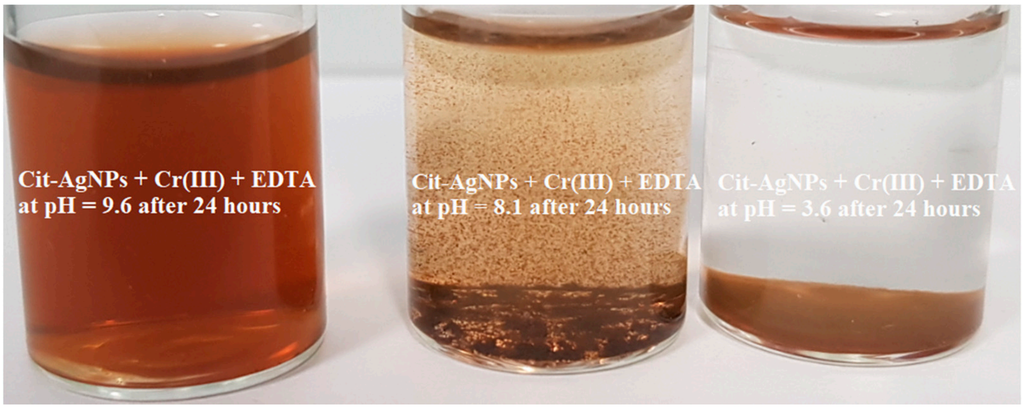


**Figure S4.** Photograph of citrate capped silver nanoparticles at different pH in absence and in presence of Cr (III) + EDTA.

The solutions used in SERS studies, were conserved at room temperatures during 24 hours in order to verify the stability of the colloidal dispersions. The colloidal system at pH 9.6 showed the highest stability among the examined pH where no precipitation was observed after 24 hours.

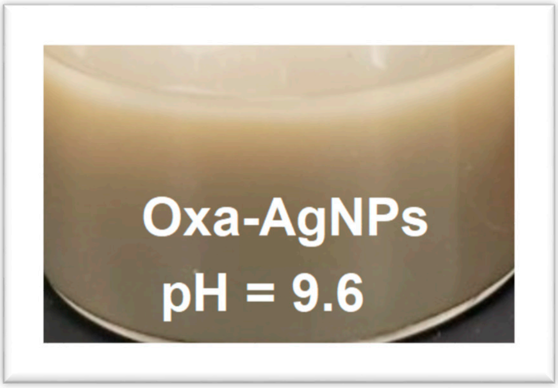


**Figure S5.** Photograph of oxalate capped silver nanoparticles at pH 9.6.

The synthesis of Oxa-AgNPs at neutral or low pH could not be successfully completed as the precipitation of the nanoparticles occurred directly after synthesis. Raising the pH to 9.6 has stabilized the colloidal dispersion. Figure S5 shows the crude Oxa-AgNPs dispersion at pH = 9.6 with a cloudy colour indicating the formation of aggregated nanoparticles during the synthesis.

**S1: Estimation of the silver nanoparticles concentration**

We have performed the SEM of the crude AgNPs in order to have an average of size distribution. All the silver nanoparticles used in this work were prepared using 1.1 mM solution of $AgNO_3$. The concentration of AgNPs is possible to be estimated from SEM images *(Johnston, R. L., Atomic and Molecular Clusters. Taylor & Francis: 2002; p 256)*. For large "spherical" metal clusters such as a silver nanoparticle, we can assume that:

$$VNP = NVatom \quad (S1)$$

$$4/3\ \pi\ (RNP)3 = N\ 4/3\ \pi\ (Ratom)3 \quad (S2)$$

where V is the nanoparticle or atom volume, R is the nanoparticle or atomic radius and N is the total number of atoms within the nanoparticle. After rearranging eq.2 we obtain:

$$RNP = N1/3\ Ratom \quad (S3)$$

For Cit-AgNPs at pH = 3.6, from the SEM result we know that RNP ≈6, and $R_{atom}$ = 0.144 nm. The number of silver atoms $N = (R_{NP} / R_{atom})^3 = (6 / 0.144)^3 = 72338$ atoms per nanoparticle.

Using the value of N, the molecular weight ($M_w$) of a single nanoparticle could be calculated from the relative molecular mass of a single silver atom ($M_{silver}$):

Mw = Msilver x N

Mw = 107.78 x 72338

Mw = 7796 kDa

Calculation of the number of nanoparticles formed ($N_{NP}$) when 1 L of 1.1 mM $AgNO_3$ is reduced by sodium borohydride was then calculated: Moles of $AgNO_3 = 1 \times 1.1 \times 10^{-3} = 1.1 \times 10^{-3}$ mol.

No. of silver atoms, $N_{atom} = 1.1 \times 10^{-3} \times NA = 1.1 \times 10^{-3} \times 6.022 \times 10^{23} = 6.6235 \times 10^{20}$

NNP = Natom / N

NNP = (6.6235 x 1020) / (7796) = 8.49 x 1016 formed per 1.1 x 10-3 mol of AgNO3

Hence the final concentration of the silver colloid, was estimated by dividing NNP by Avogadro's number (NA):

CNP = NNP / NA = 8.49 x 1016 / 6.6235 x 1023 = 1.28 x 10-8 M

The same method was applied to evaluate the concentrations of cit-AgNPs at basic pH and was found to be: 4.32x10-8 M.
